# Gender equity in hemophilia: need for healthcare, familial, and societal advocacy

**DOI:** 10.3389/fmed.2024.1345496

**Published:** 2024-04-05

**Authors:** Roberta Gualtierotti, Isabella Garagiola, Mimosa Mortarino, Silvia Spena, Olivia Romero-Lux, Flora Peyvandi

**Affiliations:** ^1^Fondazione IRCCS Ca’ Granda Ospedale Maggiore Policlinico, Angelo Bianchi Bonomi Hemophilia and Thrombosis Centre, Milan, Italy; ^2^Department of Pathophysiology and Transplantation, Università degli Studi di Milano, Milan, Italy; ^3^European Hemophilia Consortium (EHC), Brussels, Belgium

**Keywords:** hemophilia, women, bleeding symptoms, carrier, treatment challenges, psychosocial impact, quality of life, gender-specific research

## Abstract

Hemophilia is a rare bleeding disorder caused by a genetic defect on chromosome X. It is inherited as an X-linked trait, and hence, it is more frequently diagnosed in males, whereas women have been traditionally considered only as carriers of the disease. However, the role of women in families of patients with hemophilia is pivotal. As mothers, sisters, daughters, and female partners of patients with hemophilia, they play a central role in the management of the patient, considering healthcare, social, and familial aspects, but they might be affected by the disease as well, particularly in regions where consanguinity is frequent. This paper aims to explore the involvement of women in hemophilia, including their carrier status, bleeding symptoms, treatment challenges, and psychosocial impact not only related to male patients, but also as patients affected with hemophilia themselves. We advocate health equity, equal access to healthcare for men and women with hemophilia and dedicated resources to improve the unique needs of the women dealing with hemophilia, ultimately leading to improved care and quality of life.

## 1 Introduction

Hemophilia is an X-linked recessive disorder caused by the deficiency in coagulation factor VIII (FVIII; hemophilia A) or IX (FIX; hemophilia B) (1). If left untreated, patients with severe hemophilia (i.e., FVIII or FIX <1 U/dL), may experience spontaneous musculoskeletal bleeding, which account for 80% of overall bleeding events, although intracranial hemorrhage and other life-threatening bleeding events may also occur (2). Recurrent joint bleeding (hemarthrosis) occurs most frequently in ankles, elbows, and knees and if the patient is not adequately treated, this could lead to irreversible joint damage (hemophilic arthropathy). Over the last decades, the scenario of hemophilia treatment has changed dramatically, thanks to the availability of novel replacement and non-replacement drugs for prophylaxis (2).

The value of prophylaxis in decreasing the number of bleeds has been already established, although the optimal trough factor level for personalized management of patients with hemophilia is still debated. In the past, a trough level of FVIII >1% was thought to be enough to protect joints from spontaneous bleeding but Manco-Johnson et al. (3) demonstrated that patients may still experience bleeding despite prophylaxis aiming to reach this trough level. Current recommendations by the international hemophilia management guidelines, suggest a target minimum trough level of 3–5% to preserve joint function (4, 5). Nevertheless, den Uijl et al. (6) showed that patients with levels ≥10% still had, albeit very low, a risk of spontaneous joint bleeding, which was absent at levels ≥15%, findings that were recently confirmed by our group in a cohort of mild hemophilia A patients (7).

The improved availability of drugs and care pathways has increased awareness on the need of treatment also for women and girls with hemophilia (8). In X-linked recessive disorders, as males have only one copy of X-chromosome, the presence of an altered allele causes a clinically evident disease whereas females, called carriers, have a heterozygous state and a 50% chance to transmit the defective gene to a male or a female child (5). It has been described that in the family of each man with hemophilia, up to 5 potential carriers can be found and 1.6 of them are actually carriers (9). Like many X-linked disorders, the heterozygous state does not necessarily imply a disease-free state. Indeed, carriers of hemophilia may experience bleeding, especially when they have low factor levels. In the 2021 World Federation of Hemophilia (WFH) Report on the Annual Global Survey, on a total number of around 223.000 patients, the proportion of female patients was 3% for hemophilia A and 6% for hemophilia B, worldwide.^1^ In a comprehensive study involving hemophilia patients from 139 treatment centers across the United States, it was reported that approximately 0.5% of female patients had severe hemophilia, whereas 1.4 and 18% were diagnosed as having moderate and mild hemophilia, respectively (10). In addition, some carriers experience bleeding symptoms even in the presence of normal factor levels, as defined by international standards FVIII/FIX >0.40 IU/mL (8).

Despite a bleeding tendency, diagnosis and management of hemophilia carriers or female patients with hemophilia frequently remain suboptimal and personalized comprehensive care plans are rarely proposed (11). Although the gender disparity is being increasingly recognized within the bleeding disorders community, comprehensive data on women with hemophilia are still limited since they are underrepresented in surveillance databases. Most clinical trials enroll patients with severe disease, predominantly males, while moderate and mild hemophilia patients are often excluded (12). As a consequence, widespread evidence gaps exist on the efficacy and safety of different treatment options for women, as most therapies have been developed and studied primarily in a male population.

In addition, female relatives to male patients with hemophilia play an important role in their lives, being often involved in the management of the patients as caregivers. Furthermore, being carriers themselves, they must face reproductive choices, often feeling guilty, with the awareness that they might pass on the condition. This emotional burden is influenced by various factors, including personal beliefs and societal attitudes surrounding genetic conditions. Female partners of patients with hemophilia are often the patient’s caregiver but are rarely involved in dedicated programs of education on the real bleeding risk of their partners during everyday activities, including sexuality and awareness on reproductive choices.

In this brief review, we define the concepts of sex and gender, gender equality and equity, identify possible causes of health inequity for women with hemophilia and carriers of the altered allele, highlight the role of female relatives and partners to male patients with hemophilia and discuss possible solutions to promote health gender equity in hemophilia.

## 2 Defining sex, gender and gender equality

Sex and gender are conceptually different terms (Table 1). Sex is a multidimensional construct including a cluster of anatomical and physiological traits, such as external genitals, secondary sex characteristics, gonads, chromosomes, and hormones. These elements collectively distinguish organisms and contribute to the complex spectrum of biological diversity. It is typically assigned at birth by medical professionals as either male or female, based on the visual inspection of external genitals (13). However, in some cases sex traits may not fit with sex assigned at birth, as in the case of intersex individuals, who are born with biological sex characteristics, including chromosome patterns, gonads or genitals that do not fit typical binary notions of male or female bodies. In other cases, sex traits may not correspond to a single sex and may even change over time. For example, intersex individuals, which do not align with typical binary notions of male or female bodies. Therefore, sex is a complex concept that does not fit typical binary notions of male and female bodies.

**Table 1:** Definitions regarding gender equality in health.

Term	Definition
Cisgender	People whose gender identity corresponds to the sex they were assigned at birth
Cross-sex hormone therapy	Also named gender-affirming hormone therapy, involves the administration of sex hormones or other hormone-based medications in transgender people, with the aim to align their secondary sexual features with their gender identity
Gender	The social differences between men and women, which depend on what a society believes are the roles, duties, responsibilities, rights, socially acceptable behavior, opportunities and status of women and men in relation to each other
Gender dysphoria	The feeling of discomfort or distress occurring when gender identity differs from biological sex
Gender equality	Fairness and justice in the distribution of benefits, power, resources and responsibilities between women and men. It recognizes that women and men have different needs, access to and control over resources, and that these differences should be addressed in a way that rectifies the imbalance between sexes
Equality	Is about ensuring that everyone has the same opportunities and maintain their health
Equity	Is about how public services meet the different needs of the population
Equity in health	The absence of unfair, avoidable, or remediable differences in health among different population groups
Intersex individuals	People who are born with biological sex characteristics, including chromosome patterns, gonads, or genitals, which do not fit typical binary notions of male or female bodies
Sex	The different biological and physiological characteristics of men and women, such as reproductive organs, chromosomes, hormones, etc.
Transgender	Individuals with a gender identity that does not match the sex they were assigned at birth

Gender is a multidimensional construct that links a person’s individual sense of self to cultural expectations about social status, characteristics, and behavior, which may differ across social contexts and in different countries (13). Cisgender is the term that defines people whose gender identity corresponds to the sex assigned at birth. Gender dysphoria is the feeling of discomfort that occurs when gender identity does not correspond to biological sex, whereas the term transgender describes individuals with a gender identity that does not match the sex they were assigned at birth. Transgender individuals are administered sex hormones and other hormonal medications with the purpose of aligning their secondary sexual characteristics with their gender identity (Table 1). There is limited data on transgender patients with hemophilia and the effects of gender-affirming treatments in these patients. Increased awareness is crucial for fostering inclusivity and personalized management approaches for these individuals (14). This paper focuses on women and girls affected by hemophilia, recognizing that the term may not encompass all people with the ability to menstruate.

Gender equality is the concept that envisages equal consideration for women, men and gender-diverse people. In turn, gender equity is the process to achieve gender equality, by recognizing that women and gender-diverse people are not in the same starting position as men due to historical, cultural and social disadvantages (Table 1). Gender equality requires equal enjoyment by women and men of socially valued goods, opportunities, resources, and rewards. In hemophilia, possible gender inequalities that disadvantage women could be the reduced access to healthcare for carriers both with and without symptoms, with consequent limited data on these cases, reduced access to genetic counseling and the psychosocial challenges arising from the sense of guilt experienced by individuals who are carriers of a genetic defect, particularly in developing countries (15).

Recently, the European Association for Hemophilia and Allied Disorders (EAHAD) and the European Hemophilia Consortium (EHC) developed a core set of practical principles of care (16). These principles of care acknowledge equitable access and quality of care for all individuals with bleeding disorders, irrespective of gender; the need of early and accurate diagnosis of bleeding disorders in women and girls; awareness of the additional challenges female patients with hemophilia face throughout life; provision of comprehensive care in a family-centered approach; inclusion of a dedicated obstetrician and gynecologist in the multidisciplinary team; education of female patients with bleeding disorders and their families regarding the menstrual cycle and its management; early recognition and optimal management of heavy menstrual bleeding; provision of pre-conception counseling and access to prenatal diagnosis (PND); planning of a patient-centered comprehensive management throughout pregnancy and the post-partum period; improvement of involvement of female patients with bleeding disorders in registries, clinical research and innovation. Therefore, promoting gender equality in all fields, including bleeding disorders, requires a concerted effort towards identifying and rectifying power imbalances, and empowering women with greater autonomy to manage their own lives.^2^ Although only individuals can empower themselves, institutions can support the process at both the individual and collective levels. Finally, in addressing gender and health issues even in the field of bleeding disorders it is warranted to consider differences between women and men regarding age, ethnicity, geographical location, culture, education, sexual orientation, disability and socio-economic status.^3^ To support researchers in properly addressing sex and gender issues whenever designing or reporting studies and to ensure inclusivity, a set of recommendations is available: the Sex and Gender Equity in Research (SAGER) guidelines (17). The SAGER guidelines propose a list of questions that can help authors preparing their manuscript and journal editors in evaluating submitted articles, then asking authors to improve reporting of sex and gender prior to peer review, if necessary.

## 3 Carriers and female patients with hemophilia

Women and girls carrying one copy of the *F8/F9* gene with the pathogenic variant of hemophilia on an X-chromosome are named carriers. Gene variant detection is the gold standard to identify female carriers. Unfortunately, genetic testing might not be available and thus the pathogenic mutation remains unknown. Appropriate genetic tests in girls or women with FVIII or FIX deficiency should be performed even if there is no known family history of hemophilia, due to the possibility of sporadic mutations, accounting for up to 30–50% of *de novo* cases (18, 19).

Rarely, women actually experience moderate/severe hemophilia due to the inheritance of an affected altered copy of *F8* or *F9* gene on X-chromosome from both parents thus leading to the complete expression of the hemophilia variant’s severity, or one from an affected parent and the other from a *de novo* mutation, or due to X-chromosome abnormalities such as monosomy X (Turner syndrome, 45 X). Alternatively, carriers with a single affected X-chromosome may experience a skewed X-chromosome inactivation during X-inactivation, i.e., the random suppression of one of the two X-chromosomes, also known as lyonization (20, 21). In addition, a female carrier may experience abnormal bleeding even in the presence of normal factor levels, as defined by international standards of FVIII/FIX >0.40 IU/mL (22–24). Actually, factor levels are not good predictors of bleeding in these subjects (25). This may be partly due to an impaired response of FVIII to hemostatic changes (26, 27). As for FIX, an effective extravascular distribution may prevent from bleeding despite low levels of factor. Another study showed that even carriers of non-null variants could have very low factor levels and a severe clinical phenotype. Therefore, it is clear that the mechanisms that connect the type of underlying gene variant to the bleeding phenotype still need to be elucidated (21).

To improve diagnosis in women and girls with hemophilia and carriers, the International Society on Thrombosis and Hemostasis (ISTH) Scientific and Standardization Committee (SSC) approved a new nomenclature that defines five clinical categories for hemophilia carriers based on FVIII plasma levels: woman/girl with mild hemophilia when factor levels are >0.05 and < 0.40 IU/mL, moderate hemophilia when factor levels are 0.01–0.05 IU/mL, or severe hemophilia with factor levels <0.01 IU/mL. This nomenclature also considers the possibility that during the lifetime of women and girls the bleeding tendency may change. For cases with factor levels >0.40 IU/mL, the terms symptomatic or asymptomatic hemophilia carrier refer to the presence or absence of bleeding events (8).

The increased bleeding tendency in female patients with hemophilia of different severity or some carriers ranges from excessive mucocutaneous bleeding, bleeding from cuts and easy bruising, bleeding after major or minor surgery, to joint bleeding leading to arthropathy and subsequent poor quality of life or even life-threatening bleedings such as intracranial bleeds or hemoperitoneum (9, 28–32). In addition, women may experience sex-specific symptoms. The most common clinical manifestations in women and girls with hemophilia are heavy menstrual bleeding (limiting daily activities, physical exercise or social activities, lasting for more than 7 days, with the need to change tampon or pad every 2 h or less on heaviest days, or in the presence of clots), iron deficiency anemia and post-partum hemorrhages (33).

It has been shown that women and girls with hemophilia report lower quality of life compared to the general population due to the number of days of menstrual bleeding, its severity and impact on their lives (32).

## 4 Management and prevention of bleeding in female patients with hemophilia

Indeed, female patients with FVIII or FIX levels <0.40 IU/mL should be considered and managed as other patients with hemophilia, and clinicians should be aware that bleeding may also occur in carriers with FVIII/FIX levels of ≥0.40 IU/mL, with impact on their health-related quality of life.

Management of heavy menstrual bleeding envisages hemostatic agents such as tranexamic acid, desmopressin, which can be effective only in the case of hemophilia A or factor replacement therapy in more severe cases or in hemophilia B, hormonal therapies (such as oral contraceptive agents), or combinations, hormonal intrauterine devices (IUDs) and rarely surgical options (34–36). Personalized treatment options should be offered by the hemophilia care center based on age, fertility, other gynecological comorbidities, and patient’s preference, also considering cultural and psychological aspects.

The reproductive health of female patients with hemophilia requires particular attention. When planning pregnancy, a carrier or woman with hemophilia already on hormonal treatment due to heavy menstrual bleeding, should be reminded of the possibility of bleeding recurrence upon discontinuation of this medication. Adequate levels of hemoglobin and ferritin should be ensured with oral and/or i.v. iron before pregnancy.

Clotting factor replacement is necessary for invasive procedures like PND and chorionic villus sampling (CVS). During pregnancy, a physiologic increase in FVIII level occurs in carriers of hemophilia A but it should be checked repeatedly. However, in hemophilia B carriers, a rise in FIX hardly occurs (37). Differently from hemophilia A carriers, where desmopressin can be used, hemophilia B carriers with low FIX levels can only be treated with factor replacement. Due to the maternal and neonatal bleeding risk, a proper pregnancy and delivery plan should be made and shared with the patient, the partner and other members of the multidisciplinary team, possibly before 32 weeks of gestation in case preterm delivery occurs. This plan should include the evaluation of the need of factor replacement to prevent post-partum bleeding, measures to prevent bleeding in possibly affected male newborns, pain management strategies and the use of tranexamic acid in the post-partum. Regional block anesthesia is feasible when factor levels are >50 IU/dL. If clotting factor levels are <50 IU/dL at 32–34 weeks of gestation, replacement treatment is administered to avoid bleeding during and after delivery, with the aim of a through level > 50 IU/dL for around 3–5 days after vaginal delivery and 7–10 days after cesarean section (38).

Instrumental delivery is not recommended, to avoid maternal bleeding risk, and is contraindicated in the case of an affected male newborn due to the risk of intracranial bleeding. In countries with good quality of care, the risk of intracranial bleeding in the newborn with hemophilia is 3.6% i.e., at least 40 times higher than non-affected newborns and is related to the method of delivery and the mother’s awareness of being a carrier (39, 40).

Other types of bleeding and perioperative management of female patients with hemophilia should be managed based on the bleeding risk, severity of bleeding and severity of hemophilia, following the current international guidelines for hemophilia (5).

Non-replacement therapies may have a significant role in reducing bleeding complications with an easier approach compared to intravenous infusions. However, data on the efficacy and safety of these drugs in women with hemophilia are lacking (12).

We advocate for an increasing inclusion of female subjects in clinical trials, as well as the incorporation of female patients into future guidelines for the management of hemophilia.

## 5 Prenatal diagnosis and pre-implant evaluation

Women and girls with hemophilia, carriers and their partners must face important reproductive decisions, due to the possibility of having an affected child (41). Around 30% of women are unaware of their carrier status at the time of delivery, even if a positive family history is present (42, 43).

The education of future mothers is a cornerstone for quality-of-care improvement for hemophilia patients, as women play a key role in caregiving (44, 45). Even when previously aware of being a carrier, many women have described feelings of shock, sadness and grief whenever a son is diagnosed with hemophilia (46). Consequently, their family planning is often affected if the potential partner or family have limited understanding of the disease and its potential impact. This often leads to personal and familial psychosocial burden particularly in developing countries, and the consequences, such as the cancellation of marriages or divorces, can introduce instability into women’s lives (15).

An adequate genetic counseling is fundamental to support an informed reproductive decision. It is important to distinguish between information giving, education, and counseling. The purpose of genetic counseling is to provide the mother and the family with adequate information to make their own decisions regarding reproductive options and to provide support during the shared decision-making process also involving families and caregivers. Ideally, genetic counseling should be conducted before pregnancy, and for women who are already pregnant, it should be made available as early as possible. Studies investigating the experience of carriers throughout the reproductive decision-making and PND showed that the decision-making process depends on factors such as the severity of hemophilia of family members, their health-related quality of life, already having a child with hemophilia, living near a specialized medical center, having access to genetic and reproductive counseling and religious beliefs. Moreover, the cognitive and emotional aspects of this process are important as well (41, 47).

Molecular characterization, carrier detection and PND remain the key steps for planning and managing hemophilia carrier pregnancy. Current methods of PND envisage both invasive and non-invasive methods: CVS and amniocentesis are the most widely used technique for invasive PND, essentially accurate and precise methods for detecting affected males early in pregnancy. CVS could be performed between 9 and 12 weeks of gestation, thus providing early diagnosis compared to amniocentesis, which is traditionally performed around 14–16 weeks of gestation. Second trimester amniocentesis envisages that a result is available only after 17–18 weeks of gestation (48). Both techniques are considered gold standards in PND even at potential risk of miscarriage to the fetus. Miscarriage rates after CVS and amniocentesis are reported to be 0.2–0.8%, and 0.1–0.5%, respectively (49). Therefore, significant efforts have been made to obtain new starting material for non-invasive PND such as fetal cells and cell-free fetal DNA (cffDNA) present in the maternal circulation since early weeks of gestation.

During pregnancy, fetal sex determination is helpful when a specific sex is at risk of a severe genetic disease. Traditionally, prenatal fetal sex determination is performed by ultrasound, a non-invasive imaging method effective beyond 14 weeks of gestation with an accuracy >99% in cases of normal genitals (50), and by karyotype analysis following CVS and amniocentesis. Since the discovery in 1997 of cffDNA, i.e., small fragments of DNA (100-150 bp) released from apoptotic placental cells circulating in the mother’s blood, male sex determination through analysis of cffDNA has been the first non-invasive prenatal test (NIPT) developed for clinical application. NIPT is still performed in worldwide healthcare systems thanks to its advantages such as early fetal sex determination (from 7 weeks of gestation) (51), safety – it is performed by a simple peripheral blood sample from the mother -, and high sensitivity and specificity (98.9 and 99.6%, respectively) (52). Fetal sex determination by NIPT has also reduced the need of invasive procedures for the definitive diagnosis by up to 50%, since they can be avoided in case of a fetus not at risk and it is also widely accepted for detecting common chromosome aneuploidies (13, 18, 21, X and Y), large deletions and duplications, as well as the common microdeletion syndromes. The development of NIPT for the fetal gender determination has also benefited PND in hemophilia and the current WFH guidelines suggest that pregnant women who are confirmed carriers of a variant in *F8/F9* coding gene may be offered non-invasive testing to determine the sex of the fetus through cffDNA analysis in the maternal blood (5).

NIPT for sex determination relies on the amplification of specific regions (SRY, DYS14, DAZ) of the male chromosome Y.

At present, due to the poor cffDNA amount and the high contamination by maternal DNA (>90%), NIPT cannot be used for a definitive diagnosis of X-linked inherited diseases, such as hemophilia. Studies are ongoing to develop strategies for routine non-invasive PND of X-linked diseases that may replace conventional invasive procedures in the future (53–56). Actually, this process does not exclude the very low probability to give birth to a female with hemophilia.

An alternative option to conventional PND methods for couples at risk of transmitting genetic disorders to their offspring is preimplantation genetic testing (PGT), a very early diagnosis performed on embryos obtained by *in vitro* fertilization (IVF) procedures before their transfer to the uterus.

PGT consists of a genetic analysis performed using 5–10 cells obtained via biopsy from an embryo at the blastocyst stage, before transfer to the uterus. It is mostly chosen by female carriers who do not want to consider pregnancy termination, in case of an affected fetus (57). PGT is legally restricted in many countries with several legal, ethical and social implications, however over the past decade, regulatory changes in various countries in the world have shown a growing acceptance of PGT. Unfortunately, such a procedure is available only in few expert Centers in the world.

Genetic counseling should provide support, information, and assistance in making informed choices regarding family planning and reproductive options. Management of pregnancy must be carefully discussed in a multidisciplinary team involving hematologists, gynecologists, anesthesiologists and a psychologist (58).

## 6 Psychosocial impact and quality of life

Apart from the psychosocial impact of reproductive choices that were previously discussed, women and girls with hemophilia and carriers experience gender-specific psychosocial issues.

Women are still often considered as simple carriers of inherited bleeding disorders, thus often limiting their access to healthcare and to have their diagnosis acknowledged within the healthcare systems (59).

Living with hemophilia can significantly impact the psychosocial well-being of women, leading to emotional distress, social isolation, and reduced quality of life. It is well known that psychological factors may influence health decisions and that such factors, as risk perception, perceived susceptibility, perceived consequences, and confidence in one’s own physical and psychological resources might predict health behaviors.

Women with bleeding disorders often cope with their tendency to prioritize the care and interests of their children and family, downgrading their own needs. Joining a parent support group or patient organization can help provide new perspectives to face the challenges of the diagnosis while learning the importance of self-care and resilience (60, 61).

In order to increase awareness and contribute to the empowerment of women/girls with hemophilia and carriers, management should envisage psychological support since the diagnosis and discussion of the possibility of heavy menstrual bleeding and reproductive issues before pregnancy, possibly since puberty and eventually, about consequences of pregnancy (transmission of the disorder, consequences on the mother’s health status, risks at childbirth).

It is crucial that healthcare professionals of women with bleeding disorders provide education to increase awareness on the incidence, severity, and impact of bleeding disorders not only to women affected and their families, but also to the medical community. In particular, obstetricians and gynecologists are often the first specialists that patients refer to when reporting prolonged bleeding. Education programs should also be extended to the hemophilia community, so that female relatives of patients with hemophilia can be offered the opportunity of carrier testing and counseling, and treatment for their bleeding as well (2). Hemophilia treatment centers should develop and enhance services to meet the physical and psychosocial needs of women with bleeding disorders and to make these services widely available.

Contrary to historical belief, female and male patients with hemophilia experience similar non-gender specific bleeding symptoms. Among these, musculoskeletal bleeding is increasingly described in women, and joint bleeding was reported in up to 9% in several studies (62–65). Male patients with hemophilia are at risk of fractures, low bone mineral density (BMD), osteopenia and osteoporosis. The pathophysiology of low BMD in hemophilia still needs to be elucidated, being possibly due to a multifactorial mechanism including functional impairment due to arthropathy, chronic infections and treatment and possibly a biological role of FVIII or hemostasis in general (66–69). However, data regarding the women with hemophilia and carriers are very limited and indicate a higher prevalence of osteoporosis compared to controls. This is particularly important, as female patients with hemophilia and carriers are exposed to sex-specific risk factors for osteoporosis such as menopause (70).

## 7 Female partners of patients with hemophilia

Not only mothers, but also female partners are often caregivers of male patients affected with hemophilia. Women dating patients with hemophilia often face difficulties in discussing the diagnosis with their affected partners and hemophilia related burden seems to influence many aspects of the partners’ lives, in particular in family and relationships. The partner’s quality of life is directly influenced by the patient’s health (71).

Patients with hemophilia may have psychosocial problems such as depression, impaired relationship with the healthy partner or difficulties in sexual functioning with partner (72–74). In everyday life, even intimacy may be affected in patients with hemophilia, due to several physical problems such as hemophilic arthropathy leading to disability, risk of joint bleeding or hematomas of the iliopsoas or due to treatments or medications (75, 76). Many patients who received blood products before the implementation of viral inactivation procedures are affected by blood-transmitted diseases, such as human immunodeficiency virus (HIV) and hepatitis C virus (HCV) infections, further impacting intimacy and reproductive aspects (77, 78). Over the years, HIV infection has acted as an indirect source of stress for wives and partners of HIV seropositive patients with hemophilia (79).

Knowledge about intimacy, sexual health and difficulties with sexual activity in patients with hemophilia is extremely limited. In a pilot survey by Tobase et al. (80) examining sexual health 40% of patients reported that their bleeding disorder affected their sexual life. Thus, sexual health in this population requires greater attention (81, 82). Knowledge about sexuality in patients with hemophilia is important to inform physicians and stakeholders involved with policy development and comprehensive hemophilia care. Recommendations and guides for sexuality for patients with hemophilia are provided by patient associations.^4^

It is advisable that physicians and psychologists at the hemophilia treatment center also discuss these aspects of social lives with patients with hemophilia by also involving their partners, with the ultimate goal to improve health and well-being.

## 8 Discussion

The disparity of healthcare access for women with hemophilia and gender-specific issues are gaining growing attention in the community of hematologists taking care of bleeding disorders.

Although further efforts are needed to fully report annual bleeding rates and long-term complications and to evaluate quality of care in women with hemophilia and carriers, the adoption of standards that address symptom recognition is warranted, acknowledging for example that women can experience joint bleeds and pain due to arthropathy, and investigating the possibility of an increased risk of low BMD due to low FVIII or FIX levels.

Another critical issue for the future is the inclusion in trials and registries, not only of women and girls with hemophilia, but also of transgender individuals to collect as much evidence as possible and to inform decision in these cases. Finally, gender equality can be reached by also involving female partners of male patients with hemophilia in the discussion of health and psychosocial issues of these patients.

Collaboration between healthcare providers, researchers, and patient advocacy groups is crucial to enhance the care and support available for women with hemophilia, carriers and the female figures that support management of male patients with hemophilia. Similar action is required not only for women affected by hemophilia, but also for all women affected with bleeding disorders.

We advocate a paradigm shift in understanding and addressing the needs of this previously overlooked population and engaging a broader community to continue discussing, learning and taking actions that will help achieve a more equitable and inclusive society.

We aim to an improvement of health equity, equal access to healthcare for women and men with hemophilia and other bleeding disorders and dedicated resources to improve the unique needs of the women dealing with such diseases ultimately leading to improved care and quality of life.

We emphasize the importance of recognizing women as more than carriers and advocate for gender-specific research, improved diagnostic strategies and tailored treatment options. By acknowledging and addressing the unique challenges faced by women with hemophilia, healthcare professionals can optimize care and improve the quality of life of these individuals.

## Author contributions

RG: Conceptualization, Investigation, Methodology, Writing – original draft, Writing – review & editing. IG: Conceptualization, Investigation, Writing – original draft, Writing – review & editing. MM: Conceptualization, Investigation, Writing – original draft, Writing – review & editing. SS: Conceptualization, Investigation, Writing – original draft, Writing – review & editing. OR-L: Investigation, Writing – review & editing. FP: Conceptualization, Resources, Supervision, Writing – review & editing.

## Footnotes

^1^ https://www1.wfh.org/publications/files/pdf-2324.pdf

^2^ https://www.unfpa.org

^3^ https://www.paho.org/en/topics/gender-equality-health

^4^ https://hemophilia.org.uk/wp-content/uploads/2020/01/sex_BD_web.pdf
